# Home nasogastric tube program for NICU infants: a seven year retrospective outcome analysis

**DOI:** 10.3389/fped.2025.1499482

**Published:** 2025-04-24

**Authors:** Alexa Weninger, Mikayla Sabella, Abe E. Sahmoun, Mohamed W. Mohamed, Brennan Forward, Carrie M. Brower-Breitwieser

**Affiliations:** ^1^ Department of Pediatrics, University of North Dakota School of Medicine and Health Sciences, Grand Forks, ND, United States; ^2^ Department of Internal Medicine, University of North Dakota School of Medicine and Health Sciences, Grand Forks, ND, United States; ^3^ Department of Pediatrics, Neonatal Intensive Care Unit, Sanford Children’s Hospital Fargo, Fargo, ND, United States; ^4^ Department of Pediatrics, Sanford Children’s Hospital Fargo, Children’s Feeding and Nutrition Center, Fargo, ND, United States

**Keywords:** nasogastric tube, neonatal intensive care, home enteral feeding, gastrostomy tube, neonatal feeding

## Abstract

**Objectives:**

The goal of this study was to assess the safety and effectiveness of a Home Nasogastric Program for infants admitted to a Neonatal Intensive Care Unit (NICU).

**Study design:**

We performed a retrospective chart review of infants discharged from a Level III NICU to a Home Nasogastric (HNG) follow-up clinic from December 2014-February 2022. Data was recorded for two years post discharge from the NICU.

**Results:**

83 infants were included in this study. There were no emergency department visits related to feeding tube dysfunction or nasogastric (NG) tube equipment malfunctions. The number of days the NG tube was used median (IQR): 3 (2–10). Seventy-seven (93%) infants had no documented feeding problems at the end of the follow-up period. We estimate 556 hospitalization days avoided resulting in cost savings.

**Conclusion:**

The Home NG program was safe and effective. This program decreased length of NICU stay and health care associated costs.

## Introduction

The attainment of full oral feeding traditionally stands as a pivotal milestone that neonates must accomplish to qualify for discharge from the neonatal intensive care unit (NICU) (1), which at times can result in an increased length of stay. Home nasogastric (HNG) tube feeding programs were developed to assist infants in their feeding development outside of the hospital environment. Infants are able to learn oral feeding at their own pace and in a less stimulating environment compared to the hospital setting (2). Studies have shown the safety and utilization of HNG programs not only for pre-term infants, but also for medically complex infants as well (3–5). Infants utilizing the HNG program were able to reach oral feeding more quickly when compared to infants who received gastrostomy tubes in NICU (6). Other studies have also demonstrated longer duration of breastfeeding for infants discharged to a HNG tube program (7).

Recent studies have demonstrated the negative effects of prolonged NICU stays. These adverse effects cover a wide scope of an infant's care. Specifically, the hospital environment increases the risk of iatrogenic harm, as it increases the amount of exposure the neonate has to harmful technical and sensory stimuli (2, 8, 9). Avoidance of prolonged NICU admission and additional hospitalizations decreases the risk of iatrogenic harm and costs for families and hospital systems (4).

Aside from reducing adverse effects of the NICU environment, the HNG program has its own benefits. Families who have infants at high risk for gastrostomy tube placement have the ability to work toward full oral feeding at home for several weeks before making the decision to undergo gastrostomy placement. Parental satisfaction with a HNG program is very high since many experienced lower stress levels and increased familial bonding without deprivation of an authentic biological environment (10). Recent studies have found that parents who participated in the HNG program were likely to recommend the program to other parents (4, 10). Lastly, parents felt prepared and confident taking their infant home without an increase in anxiety among caregivers (11, 12).

Although there are many benefits to HNG programs, there are potential associated risks and challenges that may prevent hospitals from considering the development of a program. There is risk that the ng tube may not be used correctly in the home setting. Although caregivers are trained to use the HNG tube and pump, errors can occur that could lead to potential risk of harm to the infant. In addition to potential risk of harm to an infant, there is burden on the healthcare system to devote time and workforce to developing and maintain a program. There needs to be dedicated clinic space and staff, as families that discharge to HNG programs need to have regular access to outpatient providers and nursing support that can safely and effectively manage any needs that arise, in addition to having regular clinic visits dedicated to manage these infants while they have their NG tubes at home (5). It is important to note that the staffing and space needs required to manage infants with HNG tubes in the home setting is significantly less that the staffing and space needs required to manage the same infants in the NICU setting. Additionally, HNG programs could be absorbed into already established outpatient multidisciplinary feeding clinics or NICU follow-up clinics instead of creating a new clinic. Despite these potential concerns, data from previous studies did not reveal an increase in acute care encounters and no decrease in healthcare-related quality of life for the family (5).

There is currently a paucity of studies that have examined a standardized process for early discharge programs. We investigated safety events that occurred after discharge with the HNG, as well as data regarding length of stay in the NICU, duration of HNG tube use, feeding problems in later childhood (need for G-tube or supplemental nutrition after discharge from the HNG clinic), and potential cost savings in a rural state.

## Methods

This retrospective chart review included 7 years of NICU infants discharged to the HNG program. We performed a retrospective chart review of infants discharged from a Level III type B NICU to the HNG follow-up clinic from December 2014 to February 2022. All encounters in the medical record, including outside system records, were reviewed. The NICU is located at a regional center with an embedded children's hospital in Fargo, ND, USA. The NICU is the highest level NICU in the state. The NICU is currently a Level IV NICU, but was classified as a Level III B during the time period this data was obtained. As a result of the change in designation, the NICU managed infants from 22 weeks post menstrual age (PMA) and older with a variety of complex medical needs. Both pediatric general surgery and pediatric neurosurgery services were available to infants in the NICU, in addition to all pediatric subspecialties. The NICU transferred to higher levels of care for infants requiring cardiac surgeries or Extracorporeal Membrane Oxygenation (ECMO).

The HNG clinic was developed in order to discharge infants from the NICU with close follow-up and monitoring of the HNG tube and weight gain. The HNG clinic was also developed in a response to concerns raised by primary care physicians about the clinic resources required to safely manage infants with HNG tubes in their homes. Not all primary care clinics have staff that have the training or resources to manage HNG tubes without the use of urgent care or emergency medicine resources. The development of a formal HNG clinic provided families the necessary care and support to safely manage their infant's HNG tube and feeding in the outpatient setting. The HNG clinic was part of Pediatric Feeding and Nutrition Center housed within a pediatric subspecialty clinic. The HNG clinic was staffed by members of the multidisciplinary enteral tube feeding team, which manages all enteral feeding tubes for pediatric patients in the outpatient setting. Team members included a pediatrician, a pediatric psychologist who specializes in neonatal feeding and development, speech therapist, pediatric dietitian, and nurse navigator.

### Intra-NICU process

Infant-driven feeding practices were used in this NICU. All nursing staff had been trained using The Infant-Driven Feeding® (IDF™) Program. Oral feedings were initiated after the infant scored a 1 or 2 on The Infant-Driven Feeding® (IDF™) Scale. Nursing staff began scoring infants on the measure at 32 weeks PMA. Infants who achieved successful bottle or breast feeding that constituted at least 40% of the infant's daily volume goal were then referred to the HNG clinic for consideration for early discharge with a HNG.

### Discharge process

**Figure 1:**
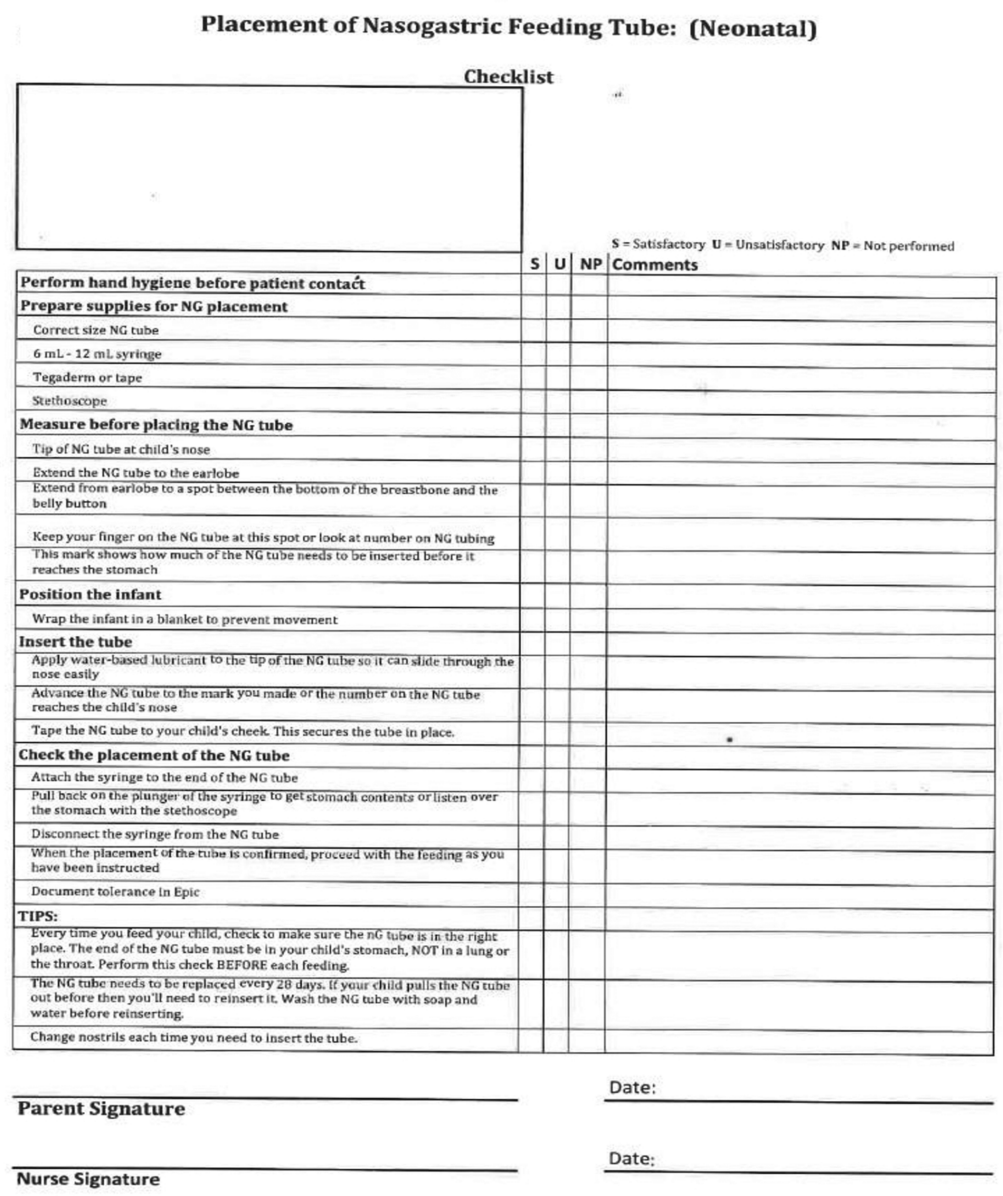
Home NG training checklist used by nursing during parent training.

Once the referral was made a HNG clinic provider reviewed the infant chart to determine if they family met inclusion criteria (Table 1). If the family met criteria for inclusion, the HNG provider met with the family to discuss the program. If the decision was to continue with the HNG program, the discharge coordinator was contacted to begin the training process. Parents received training on the use of the feeding pump by the local health care supply company. This occurred on the unit the day before discharge. Nursing staff completed training on HNG tube use and replacement using the unit HNG training checklist (Figure 1). After the training, the family was required to demonstrate their proficiency in NG tube insertion, care, and feeding pump management. The family received a feeding log and were instructed in the use of the document. Families were asked to record the timing of each feeding, in addition to all oral and HNG feeding volumes. The infant was also required to have a hospital system based primary care provider for the duration of the time the HNG tube was in place. This was a requirement at the time of program development, as many rural communities did not have access to an electronic medical record, which made shared patient care more challenging. This requirement has since been removed after the majority of neighboring health systems began to utilize an electronic medical record. While the HNG tube was in place, infants and the family were scheduled with weekly outpatient clinic visits.

**Table 1:** Inclusion and exclusion criteria for discharge with HNG program.

Inclusion criteria	Exclusion criteria
• Attend outpatient HNG visits on a weekly basis (in person or telemedicine) • Complete training on feeding pump and NG tube use. • Oral feeding 40% of total daily volume • Respiratory stability, defined as 2l or less of supplemental oxygen • Stable weight gain • Minimum of 35 weeks PMA	• Parent history of substance abuse • Parent history of significant mental health impairment • Parent history of significant neurodevelopmental disorder of significant intellectual disability • Involvement with Child Protection Services

### Post-discharge

Regular in-person weekly follow-up with the HNG clinic was scheduled and allowed for monitoring of feeding skill development, weight gain, feeding supply orders, and tube maintenance. The visits were scheduled while the infant was using the HNG tube, as most rural outlying providers were not comfortable managing HNG feedings or HNG equipment in their primary care clinics. These visits were scheduled prior to discharge from the NICU, and occurred in clinic or via telemedicine for families living outside of the metro area. Parents brought a feeding log for the team to review, which included information about each feeding, oral (bottle vs. breast) and HNG tube feeds, in addition to data on NHG tube malfunctions and tube replacements. An oral feeding was conducted with the infant, and modifications were made if indicated. Assessment of caregiver well-being was conducted by the psychologist, and appropriate referrals were made if needs arose (e.g., referral to behavioral health for significant postpartum depression symptoms, trauma symptoms related to the NICU admission, etc.). Families were able to call the clinic or send a patient message using the electronic medical record if there were questions or concerns in between visits.

Data was collected on several variables. These variables included gestational age at birth and discharge from the NICU, frequency of emergency medicine or urgent care visits associated with the feeding tube, potential negative events related to HNG tube use (tube malfunction, accidental removal of a tube while a feed was running, or incorrect tube insertion with tube use), the number of days the HNG tube was used in the home setting, as well as future g-tube placement or documented feeding difficulties. Data was obtained from the medical record from birth until two years following removal of the ng tube. Infants who were lost to follow-up after discharging from the HNG clinic were excluded from data analysis. Data was obtained from the hospital electronic medical record (EMR), as well as review of outlying hospital records that were available for review within the hospital EMR. Families were asked to report all outside hospital/clinic visits while the infant was enrolled in the HNG program. Descriptive statistics are presented as proportions (percentages) for categorical variables or median (interquartile) for continuous variables. Analyses were performed using SAS V9.4.

#### Health care saving costs

Hospital days saved was calculated by determining the total number of days the HNG tube was used in the home plus two additional days. The two additional days were added to adhere to the guidelines used in the NICU which require that infants remain admitted for 48 h after the HNG tube removal to ensure adequate feeding and weight gain. Cost savings was assessed utilizing data from a previous cost analysis completed within this specific health care system. The insurance charge for one month of HNG supplies was $189.54. The charge for one outpatient HNG clinic visit was estimated at $552, which included provider charges and associated facility fees. The charge for hospital services in the NICU was $3,181.67 per day. This daily amount includes the neonatologist daily charge in addition to the room charge. This is a rough estimate that does not capture all specific cost savings associated with each particular patient (e.g., specialty provider charges, etc.), but is used to demonstrate the potential for cost savings in the healthcare system.

## Results

### Discharge characteristics

**Figure 2:**
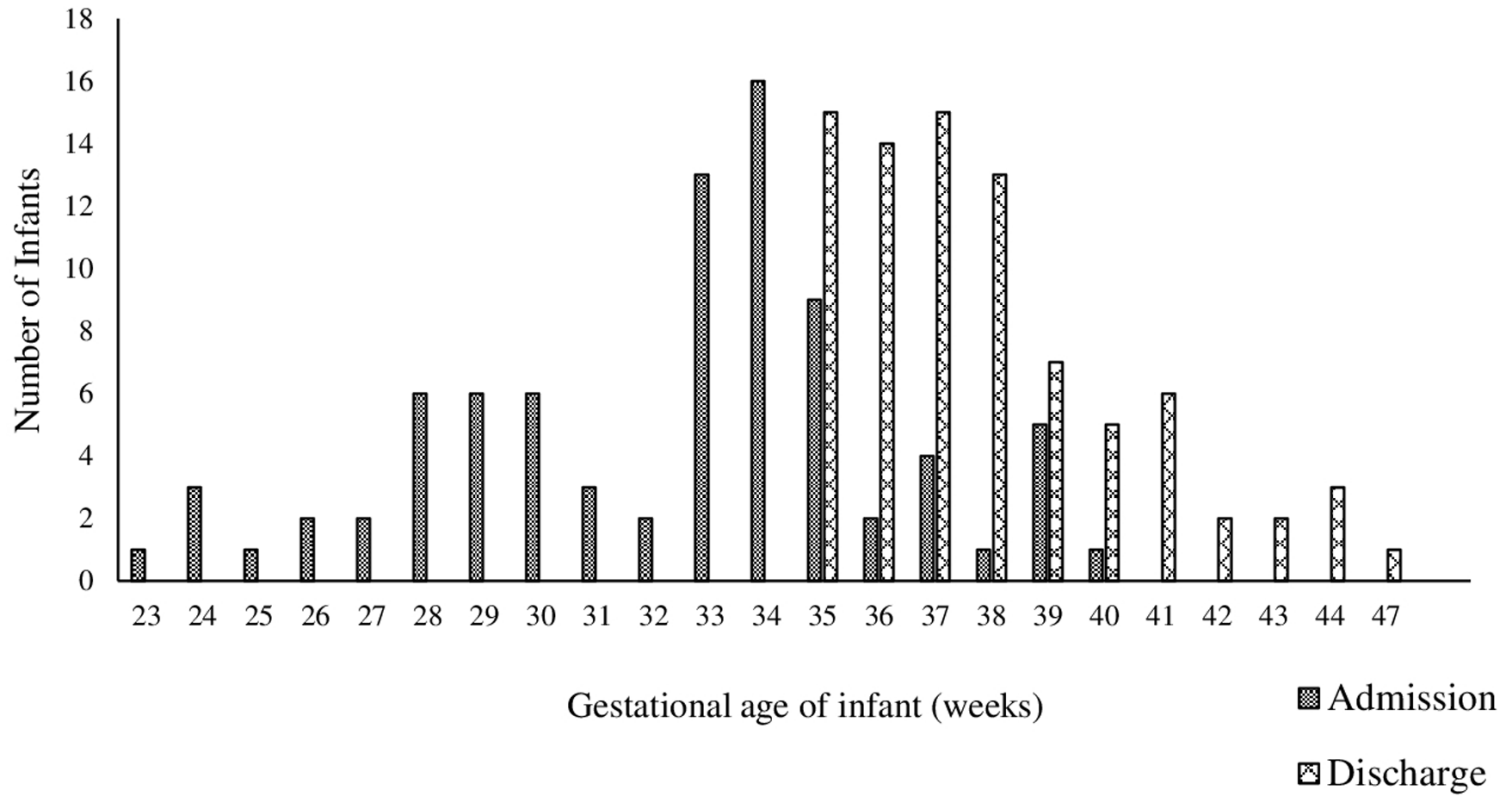
This figure visually displays the gestational age at birth and discharge for infants who discharged with the Home NK program.

A total of 83 infants met the inclusion criteria. Forty-two (51%) infants were male, and 41 (49%) were female. Thirty-eight (46%) infants were born between 33 and 35 weeks post menstrual age (Table 2). Fifty-seven infants were discharged from the NICU before 39 weeks gestation (Figure 2). The majority of infants initiated oral feedings by 33 weeks PMA (IQR 33, 35).

**Figure 3:**
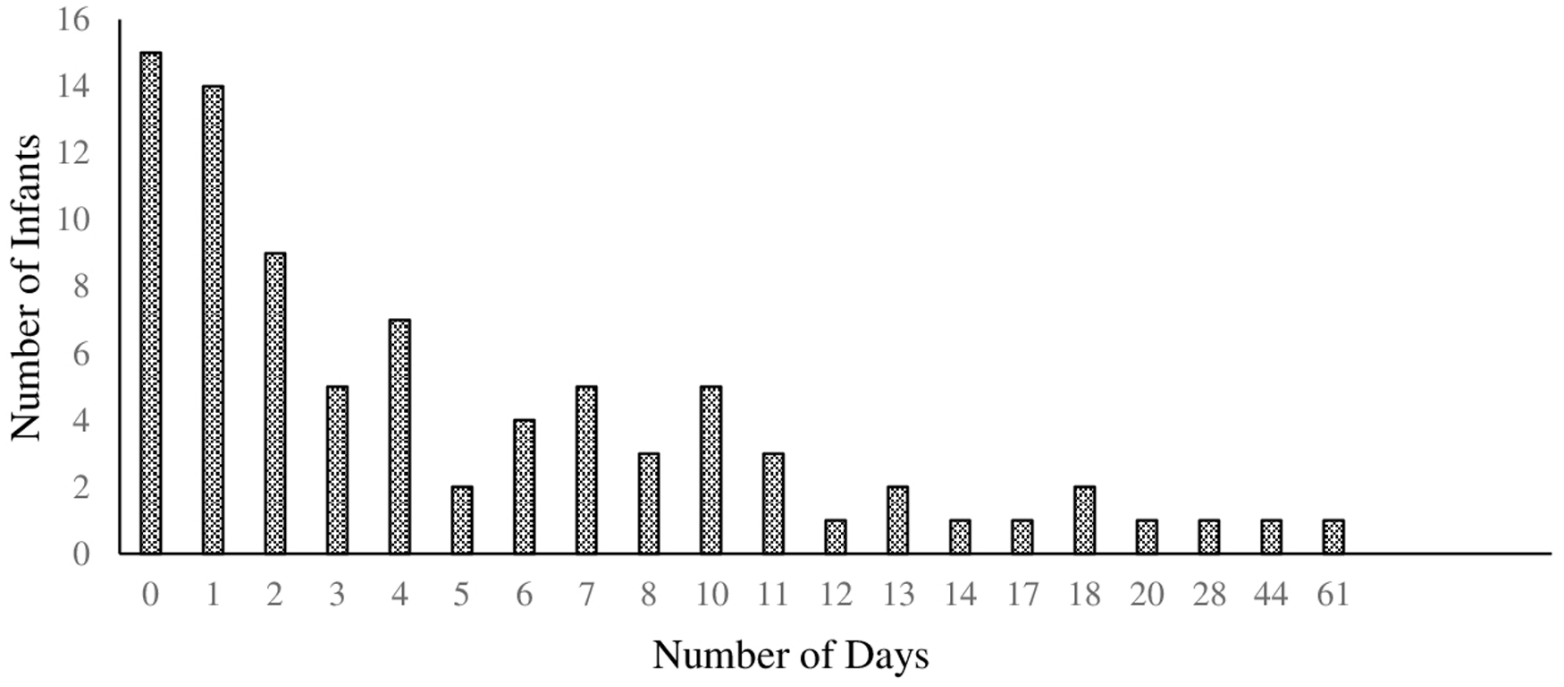
This figure details the number of days NG tubes were used throughout the Home NG program. This reveals many of the infants (74%) dif not use their NG past 8 days.

**Table 2:** Demographic variables for patients discharged with HNG program.

Variables	*N* (%)
Male	41 (51%)
Postmenstrual age at birth median weeks (IQR)	33 (29–35)
Postmenstrual age at discharge median weeks (IQR)	37 (36–39)

### Safety characteristics

Of the 83 infants included in the study, there were no emergency department visits related to feeding tube dysfunction after discharge (Table 3). Additionally, there were no documented urgent care visits related to feeding tube dysfunction or replacement. There was no malfunctions or failure of equipment (pump errors, tubes breaking, tubes clogging).

**Table 3:** Two year post discharge HNG program clinical and safety outcome data for 83 infants.

Variables	*N* (%)
ED visits	
None	83 (100)
Tube malfunctions	
None	83 (100)
Urgent Care Visits	
None	83 (100)
Readmission	
None	83 (100)
G-tube Placement	
None	80 (97)
Frequency of HNG Clinic Visits	
1	59 (71)
2	17 (20)
3	3 (4)
4	3 (4)
5	1 (1)
Number of days ng tube used at home median (IQR)	3 (1–8)

### Post-discharge outcomes

Fifty-nine (71%) infants were discharged from follow-up after one clinic visit, as they had attained full oral feeding between NICU discharge and the first clinic visit (Table 3). Eighty (96%) infants were able to obtain full oral feeds and did not require placement of gastrostomy tube. The three infants that went on to have a gastrostomy tube placed did so within 6 months of discharge from the NICU setting. Seventy-seven (93%) infants had no documented use of supplementation nutrition, ICD-10 diagnosis of a feeding difficulties, or referral for outpatient feeding therapy at the end of the two year follow-up period. There were no life-threatening events related to feeding tube use, such as aspiration related to incorrect HNG tube placement or breaking of the HNG tube while in use.

Among those who utilized the home HNG tube, the median (25th–75th) duration of HNG tube usage was 3 (1–8) days. The distribution of the number of days HNG tubes were used throughout the HNG program is shown in Figure 3. Following discharge from the NICU, there were 15 (18%) infants who did not use the HNG tube at all.

### Cost-savings outcomes

Earlier discharges were achieved for infants discharging to the HNG Program. The median length of stay was shortened by 5 hospital days (IQR: 1–8). We estimated that the median potential cost savings from earlier discharge was calculated to be $15,1661.81 per patient (IQR $8,803.47-$31,575.16) (Table 4).

**Table 4:** Cost savings and length of stay.

Variable	Cost data
Single Day NICU cost (provider + room)	$3,181.67
Monthly cost of HNG Supplies	$189.54
Daily Cost of HNG Clinic Visit	$552.00
Days HNG Used After Discharge-Median (IQR)	3 (1–8)
Estimated Length of Stay Savings per HNG Patient-Median (IQR)	5 (3–10)
Estimate Cost Savings per HNG Patient-Median (IQR)	$15,1661.81 ($8,803.47–31,575.16

## Discussion

We found that the HNG program was safe and did not result in any negative medical outcomes, such incorrect HNG placement which would result in feeding into the lungs (3, 4). There were no emergency visits following discharge related to HNG feeding. Given the lack of emergency and urgent care visits for tube replacements, we can conclude that HNG care was a safe strategy for achieving early discharge for infants from a Level 3B NICU setting who were medically stable and discharging to rural settings. In can also be concluded that parents were given well-directed instructions on managing HNG tube feeding and equipment. Additionally, this study identified significant cost-saving benefits to families and to the hospital. Based on retrospective chart review, at two years of age, infants that graduated HNG program did not have feeding problems reported on their problem list at their wellness visits, nor were any referrals placed for feeding assessment or intervention. This further enhances our safety evaluation of the program.

The HNG program effectively provided infants a safe option for early discharge from the NICU. The downstream effects of the HNG program include decreased iatrogenic harm, increased parental satisfaction, increased familial bonding (4), health care cost savings due to earlier discharge, improved bed access, and few unplanned emergency room or urgent care visits.

Our findings revealed an opportunity for even earlier discharge in 15 infants, as they did not use their NG tube post-discharge. These infants often quickly improved their feeding in the home setting. These infants were most often seen for one clinic visit before discharging from the HNG program. Although parents were pleased to have received an early discharge, there was considerable effort on the part of the parents and the medical team for only a few hospital days saved. The timing of discharge to the HNG program will greatly impact healthcare savings and value to families. At the onset of the creation of this HNG clinic, eligible infants were discharged to the HNG program once the infants feeding volumes were 40% of their daily goal. The volume goal was selected at that time for a few reasons. At the time of program development, the concept of early discharge without achieving full oral feedings was novel for many NICU providers and staff members. The volume goal of 40% was agreed upon by the NICU and HNG clinic providers as an initial inclusion criteria while data was collected on safety and success of the HNG program. Additionally, the HNG program was located within a multidisciplinary feeding center that was already at capacity in regards to the volume of patients that could be managed in the weekly clinic schedule. The 40% daily volume goal reduced the number of potential early discharges to the HNG program each week, thus ensuring there was enough capacity in the HNG clinic for follow-up visits and management. The multidisciplinary feeding center has expanded significantly since the time of the HNG pilot program, and now has capacity to manage multiple HNG patients on a daily basis. The requirement for the infant to be consistently taking 40% of the daily calorie goal has been removed. This change in criteria occurred after preliminary review of the safety and outcome data. Anecdotally, this has resulted in more days of HNG use for most infants, as well as an increase in the number of weekly clinic visits the infant attends.

The NICU is currently in the process of seeking Level 4 designation per the American Academy of Pediatrics standards for levels of neonatal care (13). As a result of this process, the NICU has observed an increase in the number of younger and more medically complex infants being admitted to the NICU that would benefit from an early discharge with HNG. As the HNG clinic has grown and developed, infants are now being assessed for HNG discharge at 34 weeks gestation to eligibility for potential early discharge to the HNG program. This process has taken several years, as both the NICU and outpatient clinic setting have worked together to create formal protocols for the care of HNG infants.

The HNG program is a vital tool for both healthcare systems and patients, but requires support from a hospital system to be successful. Discharging an infant from an intensive care setting to the clinic setting with enteral feedings requires a team approach. Dedicated workforce and clinic time is key, as success of HNG is likely related to the wrap-around care provided for patients and families. The HNG program employed the use of extensive bedside teaching and training for caregivers, close follow-up, and a clinic with dedicated nursing and provider time allocated for the management of these enteral feeding tubes.

### Limitations

While our findings demonstrate the efficacy of the HNG program in reducing hospitalization duration and healthcare costs, it is important to acknowledge the limitations of this study. The methodology employed was a retrospective chart review, lacking the design of a randomized controlled trial or comparison with a control group. The patient population selected for inclusion in the HNG program included parents with no history of substance use, significant mental health impairment, or involvement with a child protective services department. Therefore, the results of this study cannot be generalized to all patients admitted to the NICU. Although this was a limitation of the generalizability of this data, the inclusion criteria were selected to promote safety of the infant while establishing the overall safety of discharging an infant home with a nasogastric feeding tube. Future research should examine the utility of HNG discharge for infants excluded from this study. Families with a history of mental health or prior substance use may be at risk for greater iatrogenic harm from an extended NICU admission in comparison to families with strong support systems and coping skills. Additionally, the inclusion criteria required that the infant be fed at 40% of total required volumes prior to discharge from the NICU. It has been well established that higher percentages of oral feeding volume at discharge is predictive of successful transition to full oral feedings post discharge, with fewer days of HNG use needed to achieve this goal (5). Given this well-established finding, the 40% oral feeding inclusion criteria does result in a decreased impact of both cost savings and length of stay. Future research should examine the impact of reducing the inclusion criteria on health care dollars, length of stay, safety, and parental satisfaction.

The electronic medical record also poses a challenge in gathering data. The electronic medical record only recorded visits within the hospital system. While we were able to accurately record all data related to HNG tube use while the infant was in the care of the HNG clinic, we were not able to access long term data for three of the infants at age two. This did not allow for complete follow-up for those three infant records when assessing long term feeding disorder symptoms.

A significant limitation of this study is related to cost estimates. It is quite difficult to accurately assess cost savings associated with a HNG program. There are multiple factors that make estimating cost saving difficult, including insurance reimbursement, Diagnostic-Related Group (DRG) payment rates, and varying provider charges from specialists that are billed in addition to the daily room charge and neonatology charge per day. In addition these variables, a discharge allows an open bed for a new admission, which brings additional revenue that was not included in the rough assessment used for this paper. In the clinic setting, HNG discharges will result in a weekly or bi-weekly provider charges from clinic visits. The reimbursement from these clinic codes also varies based on insurance type. It becomes even more difficult to put a number on the value that an early discharge may bring to families. While there is reduced cost of inpatient health care, there is also reduced costs of childcare for other children in the home and reduced travel costs to and from the hospital are two examples of variables that cannot be accurately quantified. While the numbers used to assess cost savings are quite simplistic and may largely be an underestimate of the total cost saving associated with the HNG program, the combined value of early discharges for patient and their families with healthcare cost savings makes the HNG program a valuable resource for both hospitals and patients.

### Strengths

This is one of the few studies that have investigated safety, effectiveness, and cost savings of the HNG program in a Level 3B NICU. The NICU services the rural state of North Dakota and the surrounding rural communities of Minnesota and South Dakota. Furthermore, this retrospective chart review included data over seven years of NICU follow-up with infants discharged to the HNG program.

In conclusion, we found that our HNG program is safe and effective. Furthermore, this program can significantly decrease length of NICU stay and health care associated costs.

## Data Availability

The data analyzed in this study is subject to the following licenses/restrictions: data sets obtained from EMR. Requests to access these datasets should be directed to carrie.brower-breitwieser@sanfordhealth.org.
